# The role of moment-to-moment dynamics of perceived stress and negative affect in co-occurring ADHD and internalising symptoms

**DOI:** 10.1007/s10803-022-05624-w

**Published:** 2022-06-08

**Authors:** Lydia Gabriela Speyer, Ruth Harriet Brown, Denis Ribeaud, Manuel Eisner, Aja Louise Murray

**Affiliations:** 1 Department of Psychology, University of Cambridge, Downing Site, Cambridge, CB2 3EA, Cambridge, United Kingdom; 2 Department of Psychology, University of Edinburgh, Edinburgh, United Kingdom; 3 Jacobs Center for Productive Youth Development, University of Zurich, Zurich, Switzerland; 4 Violence Research Centre, Institute of Criminology, University of Cambridge, Cambridge, United Kingdom

**Keywords:** Perceived stress, Negative affect, ADHD, Internalising problems, Ecological momentary assessment

## Abstract

A maladaptive response to stress in individuals with high ADHD traits may be a key factor explaining co-occurring ADHD symptoms and internalising problems. The current study investigates whether between-person differences in ADHD traits are associated with differences in the within-person moment-to-moment coupling of stress and negative affect; and whether these can explain between-person differences in internalising problems (N = 262, median-age 20). Results of a dynamic structural equation model indicated that between-person differences in ADHD traits significantly moderated the daily life coupling between stress and negative affect. Further, higher ADHD traits were associated with stronger stress carry-over and higher mean levels of negative affect. Stress carry-over and mean levels of negative affect mediated the association between ADHD traits and internalising problems.

Attention Deficit Hyperactivity Disorder (ADHD) is one of the most prevalent neurodevelopmental conditions, with an estimated 2.5% of adults experiencing clinically-significant levels of ADHD symptoms (Simon et al., 2009), however a considerably larger proportion of adults may experience subclinical levels of ADHD-related difficulties (Vitola et al., 2017). Individuals with ADHD are 6 times more likely to experience internalising problems such as anxiety or depression than individuals without ADHD (Katzman et al., 2017). While the high-occurrence rates between internalising problems and ADHD have been widely acknowledged, less is still known about potential mechanisms that underlie their co-occurrence. Gaining an improved understanding of any underlying mechanisms is of vital importance for advancing therapeutic interventions.

One mechanism that may play a crucial role in the relations between ADHD symptoms and internalising problems is stress reactivity. Stress can be conceptualised as a negative cognitive-emotional state that is caused by an individual’s perceived difficulty in managing minor or major life events (Combs et al., 2012), potentially leading to emotional distress and especially feelings of hopelessness and helplessness (Ursin & Eriksen, 2004). When encountering a stressor, a number of physiological, cognitive and behavioural changes are triggered to facilitate responding to or coping with the stressor. While certain levels of stress can thus be beneficial, chronic stress has been associated with a wide range of physical and psychiatric disorders such as cardiovascular disease and anxiety disorder (Combs et al., 2012). Individuals with ADHD are at increased risk of suffering from objectively and subjectively measured stress, at least partly reflecting greater exposure to stressful events such as academic and occupational difficulties, or interpersonal conflicts (Combs et al., 2012; Hartman et al., 2019; Hirvikoski et al., 2009; Lackschewitz et al., 2008). The evidence on abnormal physiological stress responses in individuals with ADHD however is mixed, with some studies suggesting that individuals with ADHD show similar responses to individuals without ADHD or even show a reduced cortisol stress response, particularly in the presence of co-occurring conduct disorder (Booij et al., 2018). Individuals with ADHD have further been found to take longer to recover from elevated stress levels (Lackschewitz et al., 2008) and interview studies suggest that individuals with ADHD closely link experiences of stress to emotional distress and anxiety (Öster et al., 2020).

Internalising problems have also been linked to aberrant chronic stress and affective stress reactivity. In particular, research has suggested that physiological responses associated with stress (i.e., activation of the sympathetic nervous system and the hypothalamic-pituitary-adrenal (HPA) axis) can lead to increased production of adrenaline and cortisol (Cowen, 2002). Indeed, if these physiological responses do not return to homeostasis after exposure to chronic stress, an imbalance in the brain’s bio-chemistry can produce excessive levels of cortisol and changes in serotonin levels (Cowen, 2002). Imbalanced serotonin levels, in turn have been associated with depression and anxiety (Kuzelova et al., 2010). While much of the research on stress reactivity, predominantly laboratory-based studies or studies focusing solely on major life events, recent advances in ecological momentary assessment (EMA) techniques have allowed for insights into stress responses in day to day life (Joseph et al., 2021). In EMA studies, participants are asked to answer questions related to their emotional states at multiple points during the day, thus, allowing for fine-grained investigations of their fluctuations and any dynamic mechanisms that may underlie the development of mental health problems (Hamaker et al., 2018). Results from such studies have indicated that exposure to minor stressful events are associated with increases in negative affect (Jacobs et al., 2007; Joseph et al., 2021; Schlotz, 2019), with stress responses that include increases in negative affect further triggering the activation of the HPA axis as measured using salivatory cortisol levels collected at the same time as self-reports on stress and negative affect (Jacobs et al., 2007). Affective reactivity to daily life stress has further been found to be a significant risk factor for the development of depression (Booij et al., 2018).

Considering the strong links between internalising problems and experiences of chronic stress and affective stress reactivity, it is likely that the elevated stress responses associated with ADHD puts such individuals at increased risk of developing internalising problems. To date, however, little research has investigated whether differences in stress response may partially explain the co-occurrence of ADHD and internalising problem symptoms. Some evidence from previous laboratory studies suggest that individuals with ADHD are more likely to report high levels of perceived stress and respond with high cortisol levels to laboratory-induced stress; which in turn is associated with higher symptoms of depression and anxiety (Hirvikoski et al., 2009). Thus, these findings suggest that differences in stress responses may be contributing to the co-occurrence of ADHD and internalising symptoms. However, laboratory-based studies are limited in their ability to provide insights into the within-person moment-to-moment processes that drive any associations between ADHD symptoms, momentary stress, momentary negative affect and internalising problems. It is therefore necessary to investigate the complex interplay of these processes in a more ecologically valid setting. Recent advances, in EMA technology and analysis methods, now allow for such investigations. In particular, the recent development of dynamic structural equation modelling (DSEM) (Asparouhov et al., 2018; Hamaker et al., 2018) allows researchers to explore both within-person differences based on intensive longitudinal data as collect in EMA studies, as well as the opportunity to explore how between person differences such as differences in ADHD traits or internalising problems relate to any observed within-person dynamics.

In the current study, using data (*N* = 262) from the Zurich Project on the Social Development from Childhood to Adulthood (z-proso) and its ecological momentary assessment sub-study (Decades-to-Minutes, D2M), we use DSEM to investigate whether between-person differences in ADHD traits are associated with differences in the within-person moment-to-moment relations of stress and negative affect. Second, we investigate whether these within-person differences mediate the relation between ADHD traits and internalising problems. We hypothesise that individuals with higher ADHD traits will show higher overall stress levels and stress reactivity and that individual differences in the moment-to-moment dynamics of perceived stress and negative affect will mediate the relation between ADHD traits and internalising problems.

## Methods

### Participants and Procedure

Participants were 262 young adults (median age 20, 101 male) who took part in the EMA sub-study ‘Decades-to-Minutes’ (D2M) of the Zurich Project on the Social Development from Childhood to Adulthood (z-proso). z-proso is an ongoing longitudinal cohort study that has been following the lives of around 1,500 children from primary school entry in 2004 at age 7 up until age 20. The z-proso cohort was recruited from an ethnically diverse underlying population using a stratified random sampling procedure. In the current study using the D2M subsample, the country of birth of participants’ primary caregivers included 39 different countries. For details on z-proso, including information on recruitment, retention, and attrition, see the cohort profiles (Eisner & Ribeaud, 2007; Eisner et al., 2019; Ribeaud et al., 2022).

The D2M sub-study was administered shortly after the age 20 wave of the main z-proso study in order to gain insights into the moment-to-moment interplay between aggression, provocation, negative affect, stress and substance use as well as their situational contexts. D2M was conducted over a two-week period during which participants were asked to fill in short questionnaires at four quasi-random times per day between 10am and 10pm on their smartphone via an application by *LifeDataCorp LLC*. For taking part in D2M, participants were compensated with up to 50 CHF depending on their level of compliance with the maximum amount given for a response rate of at least 70% over the whole study period. At the age 20 z-proso wave, participants further completed lab-based computer assisted self-interviewing surveys on a range of psycho-social factors, including ADHD and internalising symptoms which took around 80 min to complete. As compensation, participants received 75 CHF. Participants taking part in D2M were median-aged 20. Sample demographic information is available in Table 1. Ethical approval for these studies was obtained from the University of Zurich’s Faculty of Arts and Social Science’s Ethics Committee (approval number: 2018.2.12).

**Table 1:** Sample demographic information

Variable	Category	%	N
**Sex**	Female	38.7	160
	Male	61.3	101
**Primary Caregiver**	Switzerland	62.5	163
**Country of Origin**:	Other (includes 38 different countries ranging in sample size from 1–10 participants)	37.5	98
	**Range**	**Mean**	**SD**
**Childhood Socio-economic Status (based on mean International Socioeconomic Index of occupational status household scores)**	16–88	49.09	17.58

### Measures

#### Momentary negative affect and perceived stress

Momentary negative affect was measured using an abbreviated version of the negative affect subscale of the Positive Affect Negative Affect Schedule Expanded (PANAS-X) (Watson & Anna Clark, 1999). Participants were asked to rate how afraid, scared, hostile, guilty, ashamed, upset and distressed they felt on a five-point Likert scale (range: *very slightly or not at all* to *extremely).* Scores on all items were summed up to derive a total negative affect score that was reverse coded in order for higher scores to indicate more negative affect. Perceived stress was measured using a selection of four items from the Perceived Stress Scale (PSS) (Cohen, 1994) that were adapted to a momentary format and were rated on the same scale as the negative affect items. Scores on all items were again summed up and reverse coded with higher scores indicating more perceived stressed. In total, participants completed ~ 67% of prompts relating to negative affect and perceived stress.

#### ADHD traits and internalising problems

At the age 20 wave of z-proso, participants completed a self-report version of the Social Behaviour Questionnaire (SBQ) (Tremblay et al., 1991) age-adapted for the study population. The SBQ measures psycho-social functioning in five domains: aggression, non-aggressive externalising problems, internalising problems (anxiety/depression), ADHD symptoms and prosociality. The ADHD subscale was modified to be developmentally appropriate for young adults and consisted of nine items measuring both symptoms of inattention and hyperactivity/impulsivity over the past year that were rated on a five point Likert scale from *never* to *very often.* In the current sample, the ADHD subscale had good internal consistency (*omega* = 0.87). Rated on the same scale but over a period of last month, the internalising problems subscale consisted of 14 items measuring symptoms of anxiety and depression and also showed good internal consistency (*omega* = 0.92). Psychometric analyses of the SBQ have found support for factorial and criterion validity. In addition, the SBQ has been shown to be a reliable measure of moderately low to very high levels of psychopathology in the general population (Murray et al., 2017, 2019).

### Statistical analysis

To analyse the relations between ADHD traits, internalising problems and the dynamic coupling between stress and negative affect, we used dynamic structural equation modelling (DSEM) as implemented in Mplus 8.7 (Muthén & Muthén, 2018). DSEM combines three different modelling approaches: time-series analysis, multilevel modelling, and structural equation modelling (SEM) (McNeish & Hamaker, 2019).

**Fig. 1:**
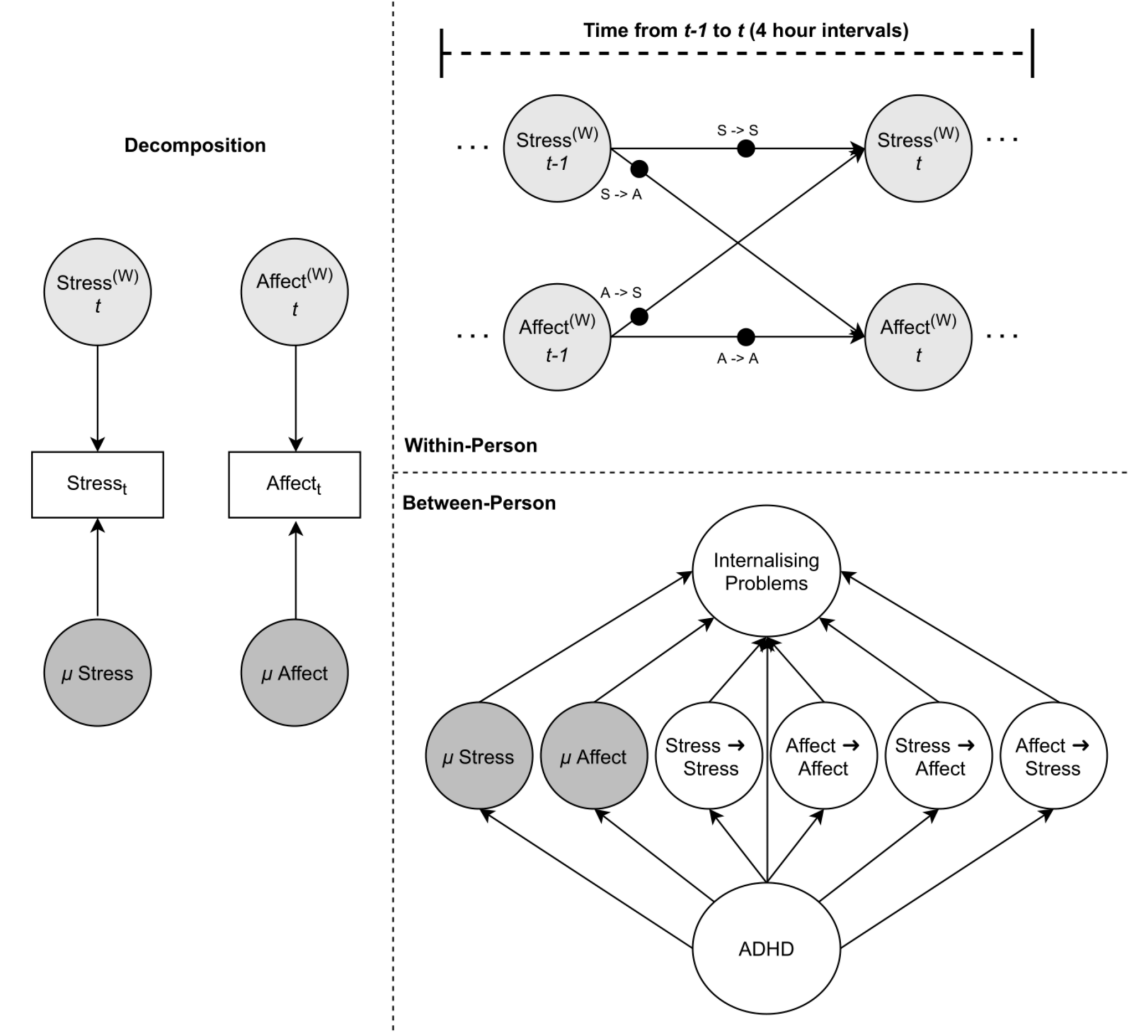
Multilevel dynamic structural equation model estimating the effect of ADHD on the dynamic coupling between momentary stress and negative affect and their relation to internalising problems. ^(W)^represents within-person estimates. Black dots indicate random effects. *µ* = Means

The model used in the current study essentially consists of three parts: First, the EMA variables stress and negative affect were modelled using a multilevel time series model that decomposed the data into latent within- and between-person components. The between-person component consists of the within-person means of stress and negative affect and the within-person component consists of the within-person temporal deviations from these within-person means (McNeish & Hamaker, 2019), thus allowing for insights into within-person dynamics while controlling for temporally stable factors such as temporally stable genetic effects or ethnicity. In the within-person part of the model, stress and negative affect at any given time-point (t) were predicted by stress and negative affect at the previous time-point (*t-1*). This allows for insights into whether stress and negative affect show significant autoregressive (i.e. predict themselves) or cross-lagged effects (i.e. predict each other). Second, we included ADHD traits as a predictor of means, autoregressive and cross-lagged effects to investigated whether between-person differences in ADHD traits significantly moderate the strength of the within-person coupling between stress and negative affect. Third, we further included internalising problems as an outcome which allowed us to investigated whether within-person differences in the relations between stress and negative affect are associated with between-person differences in internalising problem traits and to test whether the effect of ADHD traits on internalising problem traits was mediated by within-person differences in the dynamic relations between stress and negative affect. For a schematic illustration of the model, see Fig. 1.

ADHD symptoms and internalising problems were modelled using a latent measurement model using all available SBQ scores as indicators. For each latent factor, the loading for one reference indicator was fixed to one for identification and scaling. To evaluate whether the measurement model fit the data, we first conducted a confirmatory factor analysis (CFA). Next, we built a DSEM model that was run using Bayesian estimation with uninformative priors and a maximum of 50,000 Markov Chain Monte Carlo iterations, every 10th of which was recorded for estimation purposes. Once first convergence was achieved (as indicated by Potential Scale Reduction values close to 1), we doubled the number of iterations to ensure stable convergence. A Bayesian approach was used since the model includes a large number of random effects which would likely lead to convergence issues when using a frequentist framework (McNeish & Hamaker, 2019). Since participants completed EMA bursts at quasi-random times during the day, time intervals for the time-series component of the DSEM were forced to be approximately equidistant by setting time intervals to four hours with missing values specified in between observations that were further apart in time. Four hours was chosen as the optimal time interval as this resulted in the best trade-off between percent missingness introduced as a result of unequal spaced measurement occasions while allowing for a theoretically meaningful interpretation. This resulted in a total of ~ 54% missingness within the EMA data. In Bayesian estimation, missing data is treated as additional unknown quantities, thus, they are sampled from their conditional posterior distribution (McNeish & Hamaker, 2019). Simulation studies have shown that DSEM performs well with up to 90% missingness (Asparouhov et al., 2018). The model was run on Mplus 8.7 using two independent Markov Chains to reduce the chance of false convergence with the processor option set to 4 in order to utilise parallel computing to reduce estimation time. As each processor uses a different random number generator stream (resulting in a different number of iterations required for model convergence), results may differ slightly depending on the number of processor chosen (Asparouhov & Muthén, 2019). We here present the most conservative results but briefly summarise differences in results based on using 2 (the default in Mplus) or 8 processors in Appendix 1. Corresponding DSEM Mplus code and full model results including descriptive statistics are available on the Open Science Framework: https://osf.io/vgfms/.

As an additional analysis, we further adjusted the model for the following covariates: sex (female/male), primary caregiver country of origin (Switzerland/Other) and childhood socio-economic status (based on mean International Socioeconomic Index of occupational status household scores). In particular, we added these covariates as predictors of means (i.e., overall levels of stress and negative affect), autoregressive (i.e., stress→stress and affect→affect) and cross-lagged (i.e., stress→affect and affect→stress) effects as well as internalising problems in the between-person part of the DSEM model.

## Results

**Fig. 2:**
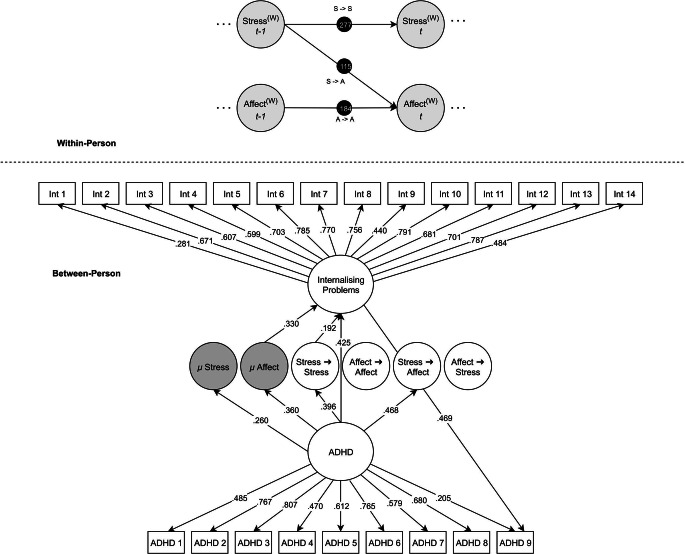
Results of the multilevel dynamic structural equation model estimating the effect of ADHD on the dynamic coupling between momentary negative affect and stress and their relation to internalising problems. Non-significant paths, residual variances, co-variances and factor variances are omitted for clarity. ^(W)^represents within-person estimates. Black dots indicate random effects. Int = Internalising Problems

A slightly modified CFA measurement model (compared with the SBQ hypothesised structure) for internalising and ADHD symptoms that included an additional cross-loading for the ‘restless inside’ item (Fig. 2) showed reasonable fit (CFI = 0.872, TLI = 0.858, RMSEA = 0.080, SRMR = 0.058, ∆BIC = 33.966) and was consequently used within the DSEM.

The baseline DSEM without covariates suggested that, at the within-person level, stress and negative affect were both autocorrelated, indicating carry-over of both stress and negative affect from one moment to the next. Momentary increases in stress were further associated with increases in negative affect over time. At the between-person level, ADHD traits significantly moderated the within-person coupling between stress and negative affect, increasing the strength of their associations. This indicates that individuals with higher ADHD traits are more likely to react with increased negative affect to a momentary increase in stress than individuals with lower ADHD traits. In addition, higher ADHD traits were further associated with stronger stress inertia, suggesting that individuals with higher ADHD traits tend to have more carry-over of stress from one moment to the next. Also, higher ADHD traits were associated with higher mean levels of both stress and negative affect. Stronger moment-to-moment coupling of stress as well as higher mean levels of negative affect were additionally associated with higher internalising problems. Finally, mediation analyses suggested a direct effect from ADHD to internalising problems, and an indirect effect via the autoregressive parameter for stress as well as higher mean levels of negative affect. The results of the baseline DSEM are summarised in Fig. 2 and in Table 2(a). Full model results are available online: https://osf.io/bz8kr/.

Results of the model including covariates suggested that the moderating effect of ADHD on the within-person coupling between stress and negative affect as well as stress inertia did not hold when accounting for sex, primary caregiver country of origin and childhood SES. Further, only mean levels of negative affect mediated the relations between ADHD and internalising problem when accounting for covariates. Finally, in addition to the significant within-person autoregressive and cross-lagged effects identified in the baseline model, results indicated that momentary increases in negative affect were also significantly associated with increases in stress. Results are summarised in Table 2(b) and available in full online: https://osf.io/hrbcg/.

**Table 2:** Parameter estimates

**(a) Baseline Model**					**(b) Model with Covariates**			
	***Estimate***	***SD***	***95% CI***_***lower***_	***95% CI***_***upper***_	***Estimate***	***SD***	***95% CI***_***lower***_	***95% CI***_***upper***_
**Means (M)**								
Stress	**3.246**	**0.186**	**2.890**	**3.620**	**2.290**	**0.354**	**1.596**	**2.986**
Affect	**5.121**	**0.306**	**4.541**	**5.750**	**4.728**	**0.430**	**3.887**	**5.570**
**Autoregressive effects (AR)**								
Stress → Stress	**0.277**	**0.021**	**0.235**	**0.316**	**0.243**	**0.022**	**0.200**	**0.282**
Affect → Affect	**0.183**	**0.022**	**0.140**	**0.224**	**0.202**	**0.022**	**0.158**	**0.242**
**Cross-lagged effects (CL)**								
Affect → Stress	0.032	0.021	-0.008	0.071	**0.056**	**0.020**	**0.021**	**0.098**
Stress → Affect	**0.115**	**0.021**	**0.076**	**0.157**	**0.093**	**0.023**	**0.048**	**0.137**
**Effect of ADHD on**								
M: Stress	**0.259**	**0.073**	**0.114**	**0.398**	**0.324**	**0.067**	**0.186**	**0.447**
M: Affect	**0.359**	**0.068**	**0.218**	**0.486**	**0.364**	**0.068**	**0.222**	**0.491**
AR: Stress → Stress	**0.392**	**0.089**	**0.200**	**0.550**	0.041	0.090	-0.134	0.218
AR: Affect → Affect	-0.245	0.120	-0.456	0.009	0.181	0.093	-0.011	0.353
CL: Affect → Stress	-0.205	0.108	-0.399	0.018	0.150	0.088	-0.029	0.313
CL: Stress → Affect	**0.464**	**0.087**	**0.273**	**0.612**	0.088	0.095	-0.099	0.271
**Effect of Sex on**								
M: Stress	**-**	**-**	**-**	**-**	**0.158**	**0.062**	**0.033**	**0.278**
M: Affect	**-**	**-**	**-**	**-**	0.114	0.066	-0.017	0.242
AR: Stress → Stress	**-**	**-**	**-**	**-**	0.134	0.081	-0.031	0.290
AR: Affect → Affect	**-**	**-**	**-**	**-**	0.032	0.085	-0.134	0.200
CL: Affect → Stress	**-**	**-**	**-**	**-**	-0.112	0.083	-0.274	0.054
CL: Stress → Affect	**-**	**-**	**-**	**-**	-0.004	0.087	-0.175	0.167
**Effect of Primary Caregiver Country of Origin on**								
M: Stress	**-**	**-**	**-**	**-**	-0.091	0.066	-0.219	0.039
M: Affect	**-**	**-**	**-**	**-**	-0.006	0.068	-0.142	0.126
AR: Stress → Stress	**-**	**-**	**-**	**-**	**0.325**	**0.087**	**0.161**	**0.469**
AR: Affect → Affect	**-**	**-**	**-**	**-**	**-0.360**	**0.081**	**-0.508**	**-0.193**
CL: Affect → Stress	**-**	**-**	**-**	**-**	**-0.300**	**0.081**	**-0.449**	**-0.132**
CL: Stress → Affect	**-**	**-**	**-**	**-**	**0.313**	**0.084**	**0.142**	**0.474**
**Effect of SES on**								
M: Stress	**-**	**-**	**-**	**-**	**0.151**	**0.071**	**0.007**	**0.288**
M: Affect	**-**	**-**	**-**	**-**	0.053	0.072	-0.090	0.194
AR: Stress → Stress	**-**	**-**	**-**	**-**	**0.411**	**0.085**	**0.234**	**0.565**
AR: Affect → Affect	**-**	**-**	**-**	**-**	**-0.376**	**0.093**	**-0.541**	**-0.176**
CL: Affect → Stress	**-**	**-**	**-**	**-**	**-0.356**	**0.086**	**-0.512**	**-0.176**
CL: Stress → Affect	**-**	**-**	**-**	**-**	**0.335**	**0.096**	**0.133**	**0.510**
**Internalising Problems predicted by**								
M: Stress	0.102	0.084	-0.062	0.271	0.151	0.092	-0.031	0.328
M: Affect	**0.331**	**0.086**	**0.149**	**0.486**	**0.270**	**0.091**	**0.088**	**0.441**
AR: Stress → Stress	**0.191**	**0.075**	**0.040**	**0.334**	**0.189**	**0.088**	**0.015**	**0.358**
AR: Affect → Affect	0.019	0.083	-0.140	0.185	0.028	0.093	-0.153	0.358
CL: Affect → Stress	0.019	0.069	-0.115	0.155	0.027	0.076	-0.123	0.176
CL: Stress → Affect	0.019	0.085	-0.146	0.184	0.053	0.087	-0.122	0.220
ADHD	**0.426**	**0.077**	**0.273**	**0.577**	**0.465**	**0.055**	**0.353**	**0.569**
Sex	**-**	**-**	**-**	**-**	**0.147**	**0.052**	**0.045**	**0.249**
Primary Caregiver Country of Origin	**-**	**-**	**-**	**-**	**0.000**	**0.072**	**-0.144**	**0.139**
SES	**-**	**-**	**-**	**-**	**-0.110**	**0.081**	**-0.271**	**0.050**
**Indirect effects: ADHD to Internalising problems** (unstandardised estimates)								
via M: Stress	0.014	0.016	-0.010	0.052	0.031	0.024	-0.014	0.023
via M: Affect	**0.067**	**0.033**	**0.022**	**0.148**	**0.063**	**0.033**	**0.018**	**0.146**
via AR: Stress → Stress	**0.041**	**0.026**	**0.007**	**0.107**	0.004	0.014	-0.022	0.035
via AR: Affect → Affect	0.005	0.026	-0.049	0.056	0.002	0.014	-0.026	0.032
via CL: Affect → Stress	-0.002	0.015	-0.039	0.024	0.001	0.010	-0.016	0.025
via CL: Stress → Affect	-0.001	0.011	-0.027	0.017	0.001	0.009	-0.007	0.023

*Note.***Bold** indicates significance based on Credible Interval (CI) not containing zero. Estimates are standardised unless otherwise indicated

## Discussion

In this study, we used an EMA design to provide insights into how ADHD traits are related to daily life dynamic relations of perceived stress and negative affect, and whether these help explain the co-occurrence between ADHD symptoms and internalising problems. Findings supported the hypothesis that ADHD traits are associated with higher stress reactivity. This was indicated by greater carry-over effects of stress as well as greater increases in negative affect as a response to prior stress among those with higher levels of symptoms. Further, higher ADHD trait levels were associated with higher overall levels of perceived stress and higher levels of overall negative affect. Further, higher stress carry-over and higher mean levels of negative affect partially mediated ADHD-internalising symptom relations. When including the effects of sex, primary caregiver country of origin and childhood socio-economic status in the model; however, the moderating effect of ADHD on stress reactivity as well as the mediating effect of stress carry-over on the association between ADHD and internalising problems were no longer significant. These results suggested that having a primary caregiver with a country of origin other than Switzerland as well as growing up in a family with higher socio-economic status was associated with increased stress reactivity. These effects appear to overlap with the effects of ADHD traits.

Our findings are in line with much of the previous literature suggesting higher stress reactivity in individuals with ADHD (Combs et al., 2012; Hartman et al., 2019; Hirvikoski et al., 2009; Lackschewitz et al., 2008). However, while previous literature relied on investigating stress reactivity in a laboratory setting, this is the first study to specifically investigate whether individuals high on ADHD traits show differences in the daily life dynamics of stress and negative affect. Our findings suggest that ADHD traits are associated with both daily stress inertia and affective stress reactivity and that the former partly explains the increased risk for internalising problems associated with ADHD symptoms. Indeed, stress inertia, or the difficulty in returning to baseline levels of stress after a stressor, may be indicative of decreased psychological flexibility (Hollenstein, 2015). In turn, decreased psychological flexibility has previously been associated with an increased vulnerability to general psychological complaints as well as onset of depressive disorder (Wichers et al., 2015). Affective stress reactivity (i.e., increases in negative affect as a response to stress) was observed to be exclusively associated with ADHD traits, but not predictive of internalising problems. This is unexpected considering that previous EMA studies have suggested that negative affect in response to minor stressors is associated with a genetic liability to depression (Wichers et al., 2007). Further research is needed to confirm these findings.

In this context, it is important to note that the effect of ADHD on increased stress-reactivity did not hold when accounting for covariates, thus suggesting that demographic factors and ADHD traits have overlapping effects on stress reactivity. Specifically, results suggested that country of origin of the participant’s primary caregiver as well as childhood socio-economic status significantly moderated not only stress inertia and affective stress reactivity but also affect inertia and stress affective reactivity (i.e., increases in stress as a response to negative affect). Individuals with a primary caregiver having a country of origin other than Switzerland and with higher SES showed increased moment-to-moment stress inertia and affective stress reactivity but decreased affect inertia and stress affective reactivity. In line with our findings, higher SES has been proposed to act as a risk factor to momentary stress in the “stress of higher status” hypothesis (Damaske et al., 2016). This hypothesis suggests that having higher status job positions leads to increased pressure to perform to high standards at work as well as conflicts between balancing work and home, in turn resulting in individuals from high SES backgrounds experiencing a greater number of stressors in daily life (Almeida et al., 2005). Such individuals have also been found to report higher levels of perceived stress as well as higher cortisol levels than individuals from lower SES backgrounds (Damaske et al., 2016). Results of the current study thus support these findings as higher SES was not only associated with increased stress reactivity but also with higher overall mean levels of stress. However, several studies have refuted this hypothesis, finding that individuals from lower SES backgrounds experience more daily life stress and low SES having been found to be associated with higher stress-reactivity (e.g., Hackman et al., 2012). Considering that SES in the current study is based on parental occupational prestige at age 7, one potential explanation for our finding of an association between higher SES and increased stress reactivity could be that young people from high SES background experience an increased weight of expectations during the transitional period of emerging adulthood, thus leading to higher stress levels and increased stress reactivity, compared to young people from lower SES background. To date, little research has investigated whether the associations between stress and SES differ based on individuals’ life stages, thus, further research on this pattern is needed. With regards to the negative effect of higher SES on affect reactivity (which has previously been associated with increased risks for developing depression; Kuppens et al., 2010), our findings are in line with prior research suggesting that lower SES is a risk factor for the development of mood disorders (Wadsworth et al., 2016).

Looking at the effect of primary caregiver country of origin on the dynamic relations between stress and negative affect, the identified significant effects suggest that there may be a cultural component to the relations between stress and negative affect. While these effects seemed to mirror the effect of SES, suggesting that caregiver’s country of origin may tap into similar factors as socio-economic status, it is also possible that the observed effects are related to differences in perceptions of stress as well as its management among individuals coming from different cultural backgrounds. Different cultures are characterised by a unique set of values, ideologies and behaviours, thus, likely resulting in different stressors but also different coping mechanisms when encountering stress (Laungani, 1993). These differences may consequently affect the moment-to-moment dynamics between stress and negative affect. To date, very little is known about the role of cultural differences in the dynamic relations between stress and negative affect as well as their relation to ADHD traits and internalising problems. Symptom presentations and perceptions of internalising disorders and ADHD have however also been suggested to differ based on cultural backgrounds (Kirmayer, 2001; Norvilitis & Fang, 2005), thus, future research on this is needed.

Future studies also need to consider other potential mediators in the ADHD-internalising association alongside stress reactivity. Findings suggested evidence only for a partial mediation effect of stress reactivity, thus, there are likely other mechanisms that further contribute to the high co-occurrence of internalising problems and ADHD symptoms. A number of studies have suggested that peer or parent-child problems contribute to the relation between ADHD and internalising symptoms (Humphreys et al., 2013; Roy et al., 2015). Also, there is some evidence to suggest that ADHD and internalising problems share underlying neurobiological and cognitive risk factors such as executive functioning deficits (Levy, 2004; Rhodes et al., 2012).

In terms of clinical implications, our results suggest that interventions aiming to reduce individuals’ stress responses may be beneficial in reducing the co-occurrence of ADHD symptoms and internalising problems. Stress management techniques are already recommended as part of occupational therapy interventions for adults with ADHD in the United Kingdom (Adamou et al., 2021). Stress management interventions such as mindfulness-based stress reduction training or cognitive behavioural therapy have been found to effectively reduce stress and improve wellbeing in individuals suffering from depression or anxiety (Carpenter et al., 2018; Chi et al., 2018; Otte, 2011), however, to date, little is known about the efficacy of such techniques for preventing the development of internalising problems as secondary to ADHD symptoms.

While this study has a number of strengths including the collection of data with high ecological validity and the application of an analysis method that allows for simultaneous investigations into within-person dynamics as well as between-person differences in these dynamics, findings also have to interpreted in light of several limitations. First, the data available for this study was cross-sectional in nature and can consequently only provide limited insights into mediation effects. Future replications using longitudinal mediation tests are therefore necessary. Future studies should also replicate these findings in population-representative and clinical populations. In addition, studies more closely investigating demographic and cultural factors are also necessary. Results of the current study suggested that demographic factors are significantly linked to the moment-to-moment relations between stress and negative affect, however, in the present study, it was not possible to gain a more in depth understanding of cultural factors that may be related to the relations between stress and negative affect as only a limited set of demographic variables was available with sample sizes also not allowing for subgroup analyses based on demographic factors. Finally, this study relied on self-reports. Considering the physiological mechanisms that are likely to contribute to the relations between ADHD, perceived stress and internalising problems, future studies should also incorporate moment-to-moment physiological measures of stress, e.g., cortisol levels into such studies (Jacobs et al., 2007). Future studies should further include a measure of emotion regulation in order to investigate whether emotion dysregulation contributes to the heightened stress reactivity in individuals with higher ADHD traits.

## Conclusions

ADHD traits are associated with increased stress reactivity which in turn partially mediates the associations between ADHD traits and internalising problems. Interventions that target individual’s responses to stress may thus be beneficial for reducing the co-occurrence of ADHD and internalising symptoms.
